# Perils of Flattery

**Published:** 1882-10

**Authors:** 


### PERILS OF FLATTERY.

Few people are proof against flattery, however broadly administered, and more have been ruined by this than, on the one hand, have been chilled by unsympathetic snubbing, or, on the other, heartened up to brave endeavor by timely praise. Indeed, there is just this difference between the two degrees of praise and flattery, that whereas the former does thus hearten up to brave and ever braver endeavor, the latter checks self-culture and destroys future progress by making you believe in attainment. According to the flatterer, the goal has been won and the great plateau of perfection reached. There are no more dreary distances to traverse, no more rugged mountain sides to climb. The place of "Rest and be Thankful" has been gained, and all that is needed now is to enjoy what you have and be grateful and glad for what you are. Like too many sweets, flattery ruins the digestion and spoils the taste for plainer and more wholesome food. No one accustomed to it can take even exhortation with a good grace; while rebuke is an impertinence to be received as an insult.

"Every one else likes me; it is only you who finds fault with me," says Araminta, half in tears of indignation, when her best friend, who loves her and sees her faults, wishes to have them corrected because of that very love. "If I were so bad as you make out, it is very odd that others should care for me!" she adds, as the demonstration which is to prove you horribly unjust when you counsel her to more reticence of manner, to more diligence and purpose in her life, to less devotion to dress and less desire for universal admiration. She is surrounded by flatterers who fool her to the top of her bent, and she cannot be persuaded that you, who have known her from her infancy, understand her perhaps a little more thoroughly than they who a month ago had not heard even her name; still less can you make her believe that the very things for which they flatter her are those most needing reform, and that characteristics which they laud as graces you are right to deprecate as dangers. That languid pose and lazy grace—some people flatter her by the hour together for the delightful refreshment of her quietness, as against the turbulent vivacity of Rosamonda! But you know that this languid pose, this lazy grace, is the outcome of an indolence which cannot meet the smallest difficulty, nor overcome the least obstacle, nor exert itself so far as to perform the most necessary duties. On the other side of the room a knot of flatterers gathers round Rosamonda, and passes its time in extol ling her spirit and smartness, her energy and life; while you, in the sorrowful recesses of your consciousness, know that all this is as purposeless and unsatisfactory as Araminta's more confessed idleness, and that neither has any other object in view than to gain the flattery which has become like the breath of their nostrils to both, and without which it seems to them that this life is in vain.

Try to inspire them with nobler views, higher aims, truer bearing, and they will turn against you—the one fretfully, the other angrily—and ask, with incredulous disdain, how it is that every one else in the world is blind but you, and so blind as to mistake black for white and good for evil? They prefer their flatterers to you, their friend. Araminta nurses her indolence. Rosamonda is satisfied with her shallowness; while the world about them extols the grace of the one and the vivacity of the other, and whispers to them cautiously that you are jealous of their social success, and annoyed because no one praises you.

Men with money, and women with beauty, are the two classes who are treated with the largest and most perilous amount of flattery. Where the calf is fat, pickings abound; and Lazarus is glad of the crumbs which fall to the ground when Dives cuts the loaf. Lavish expenditure is sure to be accompanied by a crowd of flatterers, as crows follow the plowman's furrow; for where much flows out something is to be caught, and pleasure, at least, is to be had from association with monied men who spend freely, if a practical share in the pelf itself is not to be got at. Wherefore flatterers gather round the rich man as mites multiply in the cheese, and too often he finds that—

> When the means are gone that bring this praise,
> The breath is gone whereof this praise is made.

The only way in which can be tested the sincerity of those who flatter him now as if he were a god, is by this loss of the money which attracts them. And if Timon of Athens speaks true, the loss would be a blessed one, for

> Who'd be so mock'd with glory? or to live
> But in a dream of friendship?
> To have his pomp and all what state compounds,
> But only painted, like his varnish'd friend?

and again:

> Who would not wish to be from wealth exempt,
> Since riches point to misery and contempt?

—but to misery only because they create more flattery than love—

to contempt only because they show the worst side of humanity in that shameful, shameless flattery for the goods that can be shared, rather than the good that may be loved.

As for kings and princes, they have not a chance. They are cradled in flattery as the poor are cradled in misery; and, like Alexander with his wry neck, their very defects are made occasion of praise and imitation. When quite little creatures, and long before their brains have acquired consistency or their thoughts are much beyond the level of idiotcy, they are treated with "respect" by the grown men and women appointed to guard and rear them; while they are accustomed to the loud huzzas of assembled multitudes, and their personality applauded as if they had really done something praiseworthy. Grown older, they find slaves and creatures of every form. Men of character and virtue wink at their excesses; women of repute and modest bearing forget to chide or to repulse; that fatal heritage of flattery belongs to them equally with their title, their future, their estate; and save in a country like our own, where the press is free and editors are men, not worms, they never hear the faintest breath of blame, and are substantially as much deified to-day as they were in the time of ancient Rome, when an emperor would give himself out as the son of Jupiter or the descendant of Apollo, and statues were raised to his honor and sacrifices made in his name, all the same as if he had been the god whose paternity he claimed. It is praise and flattery all through; and if "uneasy lies the head that wears a crown," it is also "the curse of kings to be attended by slaves," who, if they do not yet "take their humors for a warrant to break within the bloody house of life," yet do take their humors for a warrant of command which they are bound to obey.

And so with beauty. No sooner does a pretty, fresh young thing make her appearance in society than all conspire to flatter her and rob her of the very charm they praise. Now, she is unconscious, devoid of vanity, considerate for others, without egotism or self-seeking; in a short time she will be made the exact contrary of all this. She will have been flattered into self-consciousness, which is the first step to vanity, and that very quality of her artlessness will be lost in the cloud of incense burned to celebrate its charm. She will have learned that she has fine eyes, and every look, every expression, will have been chronicled and detailed; she will hear that she has fine hair, and she will learn its exact shade in the sun and the depth of its lustre in the shadow; and she will be told of her creamy skin, of her prett[illegible]

mouth, of her graceful figure, of her sweetness and maidenliness, and simplicity and modesty. And the consequence of all this will be that she will study her looks and her poses, her complexion and her attitudes before the glass, till not a trace of unconsciousness is left, and she becomes as much a creature of art as if she were a paid actress on the boards. She will spend hours in arranging her hair so as best to show its curl, its sheen, its length, its thickness. Before this time she had dressed it simply, by which all its beauties were half revealed, half concealed; that beauty without intention which is the most beautiful thing of all. She will spend hours in learning this trick of her head, that action of her hands; this way of standing and that way of sitting; and here again she will lose all the grace of simplicity which first attracted to her the baleful crowd of flatterers who have destroyed what they so be-lauded.

It is not only from the world without that we gather the sweet poison of flattery. We get quite enough at times at home, and are taught to believe that we are unique in our acquirements, and without rivals in our graces. The brothers of affectionate sisters are the cleverest and handsomest and most accomplished. Lina's drawings are perfection, Nina's voice would be finer than Patti's were she to sing at the opera, May's music is as good as Halle's, no one dances so well as Fay, Lily has the most splendid hair that can be seen out of a show, and Milly has eyes that would make the fortune of one whose face was to be her fortune, should chance throw her into the way of King Cophetua. So on, of all they do and are—their work, their croquet, their tempers, their brains. Only when they go out into the world and measure themselves against others do they learn truly what their own dimensions really are. The loving flattery of home has made them bigger and higher in every way, and the waking to the bare truth is sometimes painful enough. But, bad as is all this flattery from the outside, that which we give to ourselves is the worst of all. "*L'amour propre est le plus grand de tous les flatteurs*," says Rochefoucault, and the Frenchman was right. If we were flattered every day in the week, and yet did not accept as true what we were told, it would do us no harm; but when we begin to flatter ourselves we have tumbled into the pit, and getting out again is the difficulty, which not all of us are able to master. We have to be somewhat severely handled before we can say that we have learned our lesson—of how to walk in safety, free of flattery and its lures, and how to avoid the pitfalls of vanity resulting therefrom.

